# Phylogenomic analyses predict sistergroup relationship of nucleariids and Fungi and paraphyly of zygomycetes with significant support

**DOI:** 10.1186/1471-2148-9-272

**Published:** 2009-11-25

**Authors:** Yu Liu, Emma T Steenkamp, Henner Brinkmann, Lise Forget, Hervé Philippe, B Franz Lang

**Affiliations:** 1 Robert Cedergren Centre, Département de biochimie, Université de Montréal, Montréal, Québec, Canada; 2 Department of Microbiology and Plant Pathology, Forestry and Agricultural Biotechnology Institute, University of Pretoria, Pretoria, South Africa; 3 Present address: Donnelly Centre for Cellular and Bio-molecular Research, Department of Molecular Genetics, University of Toronto, 160 College Street, Toronto, ON, M5S 3E1, Canada

## Abstract

**Background:**

Resolving the evolutionary relationships among Fungi remains challenging because of their highly variable evolutionary rates, and lack of a close phylogenetic outgroup. Nucleariida, an enigmatic group of amoeboids, have been proposed to emerge close to the fungal-metazoan divergence and might fulfill this role. Yet, published phylogenies with up to five genes are without compelling statistical support, and genome-level data should be used to resolve this question with confidence.

**Results:**

Our analyses with nuclear (118 proteins) and mitochondrial (13 proteins) data now robustly associate Nucleariida and Fungi as neighbors, an assemblage that we term 'Holomycota'. With Nucleariida as an outgroup, we revisit unresolved deep fungal relationships.

**Conclusion:**

Our phylogenomic analysis provides significant support for the paraphyly of the traditional taxon Zygomycota, and contradicts a recent proposal to include *Mortierella *in a phylum Mucoromycotina. We further question the introduction of separate phyla for Glomeromycota and Blastocladiomycota, whose phylogenetic positions relative to other phyla remain unresolved even with genome-level datasets. Our results motivate broad sampling of additional genome sequences from these phyla.

## Background

The investigation of previously little known eukaryotic lineages within and close to the opisthokonts will be key to understanding the origins of Fungi, the evolution of developmental traits in Fungi and Metazoa, and ultimately the origin(s) of multicellularity [1-3]. In particular, it will help to establish which and how many developmental genes are either shared or specific to these two major eukaryotic groups. In this context, it is essential to determine the precise phylogenetic position of candidate protists that are close to Fungi, Metazoa, or opisthokonts as a whole.

The candidate organisms choanoflagellates, ichthyosporeans and *Ministeria *have been convincingly shown to be relatives of Metazoa (combined in a taxon termed Holozoa; [4]) by using molecular phylogenetics with genomic datasets (e.g., [4-8]). Yet, there are remaining questions about the exact phylogenetic positions of *Capsaspora *[5,8] and *Ministeria *[7] within Holozoa. Another, less well studied group of protists are Nucleariida, a group of heterotrophic amoeboids with radiating filopodia. Nucleariids lack distinctive morphological features that might allow associating them with either animals or fungi. Their mitochondrial cristae are either discoidal-shaped or flattened [9-11]. Indeed, initial phylogenetic analyses based on single genes have been inconsistent in placing them even within opisthokonts. There has been also confusion due to the inclusion within Nucleariida of *Capsaspora owczarzaki*, a species that is now excluded from this group and shown to be clearly associated with Holozoa [5,11-17].

Overall, the phylogenetic position of the 'true' nucleariids remains controversial. In a more recent phylogenetic investigation with four nuclear gene sequences (EF-1α, HSP70, actin and β-tubulin), nucleariids associate confidently with Fungi, but only when selecting two slow-evolving chytridiomycetes [18]. When improving the taxon sampling to 18 fungal species, the bootstrap support (BS) value for fungal monophyly drops to 85%, and alternative nucleariid positions are not rejected with the approximately unbiased (AU) test [18,19]. In this context, it seems noteworthy that *Nuclearia *and fungi other than chytrids are fast-evolving, and that the rate of tubulin evolution varies strongly among species of the latter dataset (correlating to some degree with the independent loss of the flagellar apparatus in non-chytrid fungi and in *Nuclearia*). Together, these rate differences at the gene and species levels may increase long-branch-attraction (LBA between the two fast-evolving groups) thus causing weaker support for fungal monophyly and the nucleariid-fungal sister relationship, or predicting altogether incorrect phylogenetic relationships.

These unresolved questions served as motivation for the current phylogenetic analyses that are based on broad taxon sampling, substantially more nuclear genes (available through expressed sequence tag (EST) or complete genome projects), and comparative analyses of nuclear and mitochondrial gene datasets. To this end, we sequenced several thousand ESTs each from two *Nuclearia simplex *strains (probably representing separate species based on the high level of sequence divergence between them), and added them to a previous dataset [20] along with new genome data available from Holozoa (*C. owczarzaki, Amoebidium parasiticum, Sphaeroforma arctica*; [5]) and Fungi (*Allomyces macrogynus, Batrachochytrium dendrobatidis*, and *Mortierella verticillata*). We then sequenced the mitochondrial genome of one of the two *N. simplex *strains. Similar to the nuclear genomes of fungi, their mitochondrial genomes also evolve at varying rates thereby introducing a considerable potential for phylogenetic artifacts. However, phylogenetic comparisons between mitochondrial and nuclear data provide valuable, cross-wise indicators of phylogenetic artifacts as the respective evolutionary rates differ between the two genomes. For instance, such comparisons have revealed inconsistencies for the positioning of *Schizosaccharomyces *species within Taphrinomycotina [21], and of *Capsaspora *within Holozoa [5,7,8].

If the nucleariids are indeed the closest known relatives of Fungi as claimed [18], this protist group will provide an excellent fungal outgroup that would ultimately facilitate the settling of controversial phylogenetic placement of taxa within Fungi and/or in close neighboring groups. Among the debated issues are the monophyly and appropriate classification of the traditional fungal taxa Chytridiomycota and Zygomycota. Previous analyses based on single or a few genes have been inconsistent in answering these questions, and often lack significant support [22-31]. For example, the analyses of ribosomal RNA data supports the sister relationship between Glomeromycota and Dikarya (Ascomycota plus Basidiomycota) [29], while analysis of genes encoding the largest and second-largest subunits of the nuclear RNA polymerase II supports the monophyly of Zygomycota in its traditional definition [25].

Phylogenetic positioning of the extremely fast-evolving Microsporidia (causing strong LBA artifacts in phylogenetic analyses) is another controversial issue of great interest. In some of the most recent analyses, Microsporidia have been placed either close to zygomycetes/Mucorales [32,33], or together with *Rozella allomycis *[24]. Together with environmental sequences, *Rozella *species form part of a large, diverse and relatively slowly evolving lineage (designated "Rozellida"). They branch as a sister clade to Fungi [24,34], which raises the additional question whether they should be considered to be true fungi as originally proposed [35]. Testing the above alternative hypotheses on microsporidian affinities by phylogenomic analysis will require much more data from Rozellida (a few genes are known from *Rozella allomycis*, but largely insufficient for inclusion in our analyses), and from a much wider range of the paraphyletic zygomycetes. Generation of genome-size data will be further critical for applying methods that reduce LBA artifacts such as removal of fast-evolving genes or sequence sites (e.g., [36] and references therein).

Despite these and various other unresolved phylogenetic issues, fungal taxonomy has been substantially redefined in a recent proposal [28]. Chytridiomycota is still treated as a phylum, but now include only Chytridiomycetes and Monoblepharidomycetes. Other traditional chytrid lineages such as Blastocladiomycota and Neocallimastigales have been elevated to phyla based on the analyses of LSU and SSU rRNA [23], although support with these and other molecular markers is inconclusive. In turn, the traditional phylum Zygomycota has been altogether removed from this taxonomy [28], because evolutionary relationships among its members are currently unresolved and suspected to be paraphyletic. Zygomycota are now reassigned into a phylum Glomeromycota plus four subphyla *incertae sedis *(i.e., uncertain): Mucoromycotina, Kickxellomycotina, Zoopagomycotina and Entomophthoromycotina. To revisit these somewhat contentious issues, we compared results with mitochondrial and nuclear phylogenomic datasets, and further analyzed the effect of extending fungal species sampling, with the two *N. simplex *strains as the outgroup.

## Results and Discussion

### Phylogenomic analysis with the Eukaryotic Dataset supports Nucleariida as sister to Fungi

**Figure 1:**
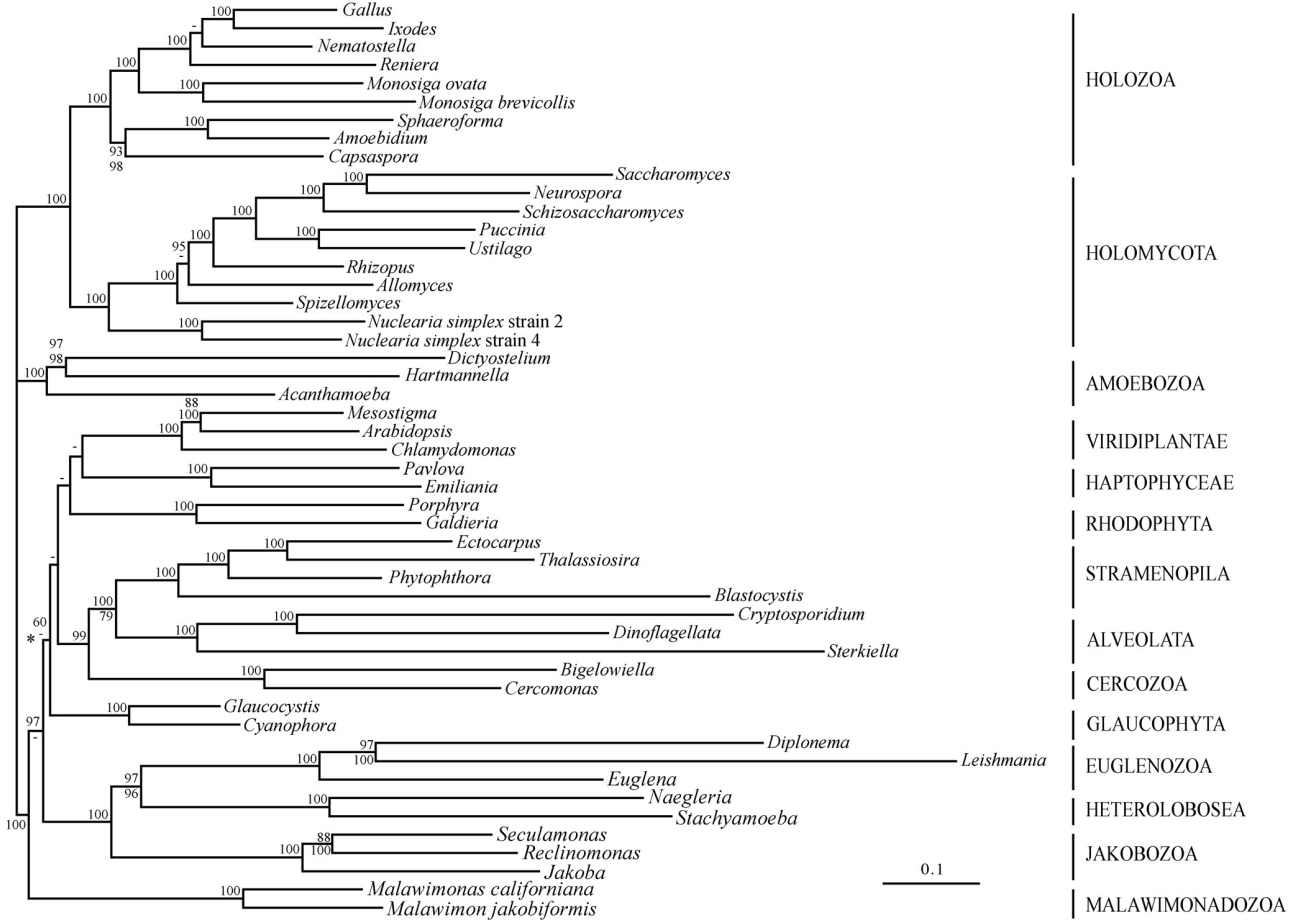
**Tree of eukaryotes based on Eukaryotic Dataset**. Trees were inferred with PhyloBayes and rooted following a previous suggestion [69,70]. The values at branches indicate bootstrap support (BS) values (upper value, BI/CAT model; lower value ML/WAG model). Values below 60% are indicated by a hyphen; when BS values are equal only one is indicated. The posterior probability values using PhyloBayes are 1.0 for all except two branches (0.98 for the branch uniting Viridiplantae and Haptophyceae; 0.90 for the clade indicted by *). The analyses using ML (RAxML, WAG+Gamma; four categories, see additional file 1) support the alternative grouping of Malawimonadozoa and JEH with a BS of 77%. Other minor differences include Plantae relationships and the placement of Haptophyceae, which receive no solid support in both BI and ML analyses.

Phylogenomic analysis of the Eukaryotic Dataset with one of the currently most realistic phylogenetic models (category mixture model (CAT); [37]) confirms the monophyly of major eukaryotic groups including Holozoa, Fungi, Amoebozoa, and Viridiplantae. Further, *Amoebidium*, *Sphaeroforma *plus *Capsaspora *form a monophyletic group, and *Nuclearia *is without a doubt the closest known sister-group to Fungi (100% BS; Figure 1). Also some higher-order relationships are recovered with significant support, such as opisthokonts and the two recently proposed supergroups JEH (jakobids, Euglenozoa plus Heterolobosea [20]) and CAS (Cercozoa, Alveolata plus Stramenopila [20,38,39]), whereas monophyly of Plantae, Excavata and Chromalveolata is not found. Evidently, the taxon sampling of protists in our dataset is insufficient for (and not aimed at) resolving the phylogenetic relationships among these latter lineages, as it was meant to constitute only a strong and well sampled outgroup to opisthokonts.

Analysis of the Eukaryotic Dataset with maximum likelihood (ML) using RAxML [40] and the commonly used WAG+Γ model generated a similar tree topology (Figure 1 and additional file 1). Deep opisthokont divergences are predicted consistently and with significant support (BS > 98%), with *Nuclearia *clearly sister to Fungi (100% BS) and choanoflagellates the closest neighbor of animals. *Amoebidium*, *Sphaeroforma *plus *Capsaspora *form a monophyletic sister group to animals plus choanoflagellates, consistent with a previous analysis [5] but contradicting others [7,8]. The reasons for this incongruence may be related to differences in data and taxon sampling. Our dataset contains 50 eukaryotic species with a close outgroup to Holozoa (i.e., including nucleariids together with fungal representatives), compared with a total of only 30 species in a more extensive previous analysis [7]. In contrast to our analysis using Bayesian inference (BI), ML associates Malawimonadozoa with JEH (77% BS), a tendency noted and discussed previously [20,41], and an issue to be addressed by better taxon sampling in this group (currently, data are available from only two species). Other minor differences between WAG *versus *CAT model analyses (yet without statistical support in favor of alternatives) are in relationships within Plantae and the placement of Haptophyceae.

We further investigated if the position of *Nuclearia *next to Fungi might be affected by potential phylogenetic artifacts, such as compositional sequence bias and/or LBA [36,42]. This is suspected because of the highly varying evolutionary rates both within Fungi and in protist outgroups, and the unusual result that better taxon sampling in Fungi reduces phylogenetic support for the *Nuclearia *position ([18]; see introduction). To do so, we first eliminated fast-evolving species from the dataset: *S. cerevisiae*, *Blastocystis hominis*, *Cryptosporidium parvum*, *Sterkiella histriomuscorum*, *Diplonema papillatum *and *Leishmania major*. The results from analyses using RAxML were essentially unchanged, both with respect to tree topology and BS values (additional file 2). To counteract sequence bias, we recoded the 20 amino acids into six groups as previously proposed [43]. Again, phylogenetic analysis of this dataset using P4 [44] generated essentially the same tree topology, with some support values decreased due to loss of information by recoding (additional file 3).

Finally, we evaluated the positioning of *Nuclearia *next to Fungi with the AU and weighted Shimodeira Hasegawa (wSH) likelihood tests [45]. For this, we compared the topology presented in Figure 1 with competing tree topologies in which the two *Nuclearia *strains were moved as sistergroup to all major eukaryotic lineages, and all possible positions within Opisthokonta. The results of both tests confirm *Nuclearia *as the closest neighbor group of Fungi, with all alternative topologies rejected at a significance level of p = 0.002 (Table 1). Given the unequivocal support for *Nuclearia *as the fungal sistergroup, we propose the term 'Holomycota' to refer to the assemblage of Nucleariida plus Fungi.

**Table 1:** Comparison of alternative tree topologies with AU and wSH tests.

Rank	Tree topology	ΔlnL	AU	wSH
1	**Best tree **(see Figure 1)	0	**1.000**	**1.000**
2	*Nuclearia *sister of Holozoa	187.9	0	0
3	*Nuclearia *sister of Opisthokonta	237.4	0	0
4	*Nuclearia *sister of Asco- + Basidio- + Zygomycetes	418.2	0	0
5	*Nuclearia *sister of *Capsaspora *+ *Amoebidium *+ *Sphaeroforma*	478.2	0	0
6	*Nuclearia *sister of Metazoa + *Monosiga*	495.3	0	0
7	*Nuclearia *sister of *Allomyces*	511.1	0	0
8	*Nuclearia *sister of *Spizellomyces*	513.7	0	0
9	*Nuclearia *sister of *Capsaspora*	534.3	0	0
10	*Nuclearia *sister of *Amoebidium *+ *Sphaeroforma*	561.2	0	0
11	*Nuclearia *sister of Amoebozoa	621.2	0.002	0
12	*Nuclearia *sister of Opisthokonta + Amoebozoa	626.8	0.002	0
13	*Nuclearia *sister of Asco- + Basidiomycetes	704.5	0	0
14	*Nuclearia *sister of *Monosiga*	727.5	0	0
15	*Nuclearia *sister of Metazoa	738.9	0	0

Log likelihood differences and AU and wSH p values of top-ranking trees are listed.

### Mitochondrial phylogeny and genomic features support monophyly of the Holomycota

Phylogenetic analyses of nuclear *versus *mitochondrial datasets are expected to come to similar conclusions, thus providing independent evidence for the given phylogenetic relationships. To this end, we sequenced and analyzed the complete mitochondrial DNA (mtDNA) of one of the *N. simplex *strains (a circular mapping DNA of 74 120 bp; see additional file 4). Note that growth of *Nuclearia *is complicated (the standard method calls for growth on Petri dishes with a bacterial lawn as food source), and that it is difficult to obtain sufficient cell material for mtDNA purification, explaining why we succeeded for only one of the two *Nuclearia *species.

The *Nuclearia *mtDNA contains a high number of introns (21 group I, and one group II), and mitochondrial protein genes appear to be translated with the standard translation code. These features are also widespread in Fungi. In contrast, Holozoa all use a mitochondrial UGA (tryptophan) codon reassignment, and contain no or only a few introns (with the notable exception of Placozoa, an enigmatic group of Metazoa [46]).

**Figure 2:**
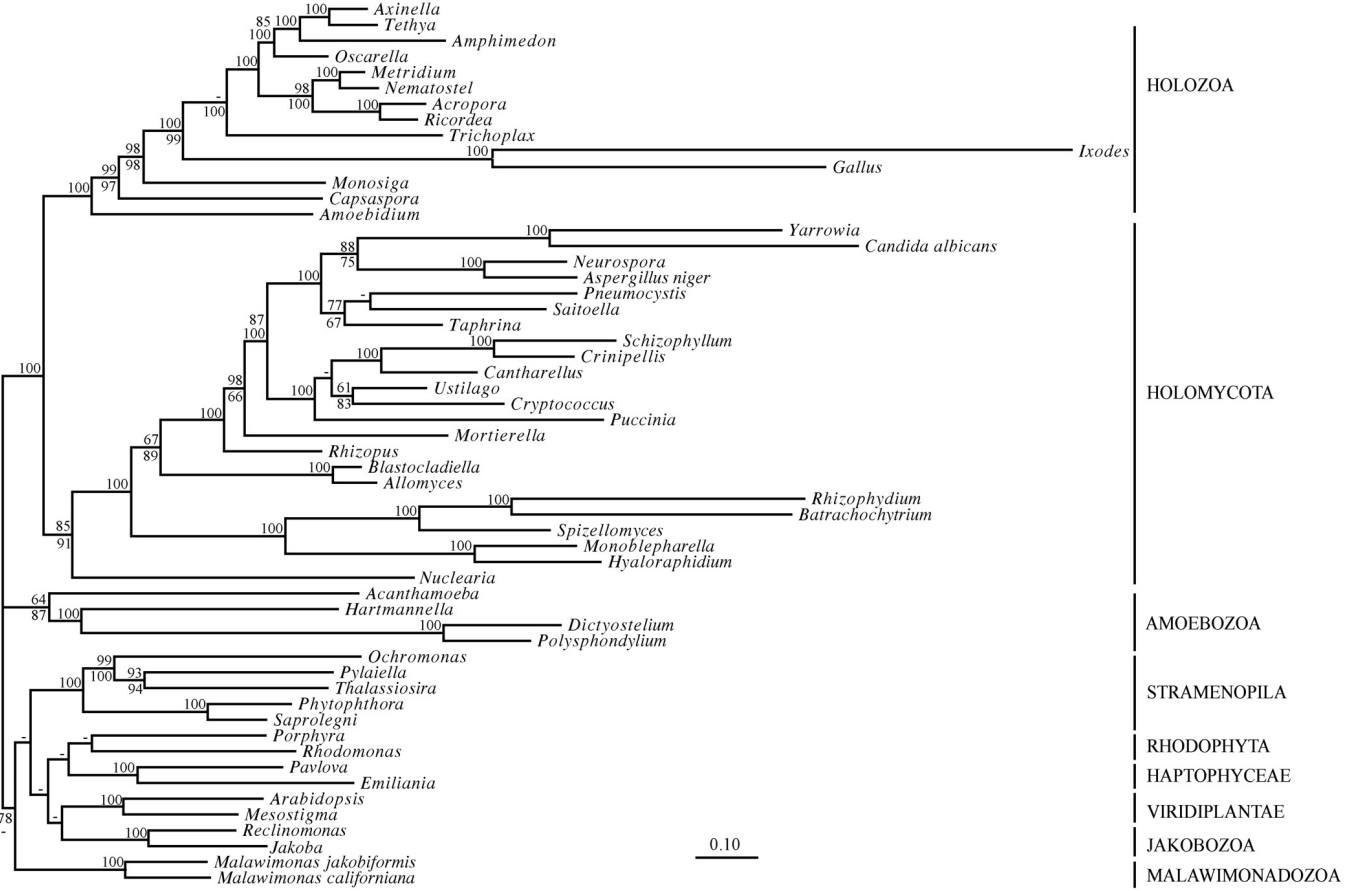
**Phylogeny inferred from the mitochondrial dataset**. For details on figure description, evolutionary models and phylogenetic methods, see legend of Figure 1. Note that as already noted in a previous publication [5], the phylogenetic position of *Capsaspora *with mitochondrial data differs from that with nuclear data (Figure 1). We attribute this inconsistency to the limited availability of mtDNA sequences from *Capsaspora *relatives, and a strong LBA artifact introduced by the fast-evolving Bilateria in concert with *Trichoplax*. Further, the placements of *Cryptococcus *and *Ustilago *differ (although without significant support) from those with nuclear data (see Figure 4), although results with the much larger nuclear dataset are more likely to be correct.

**Figure 3:**
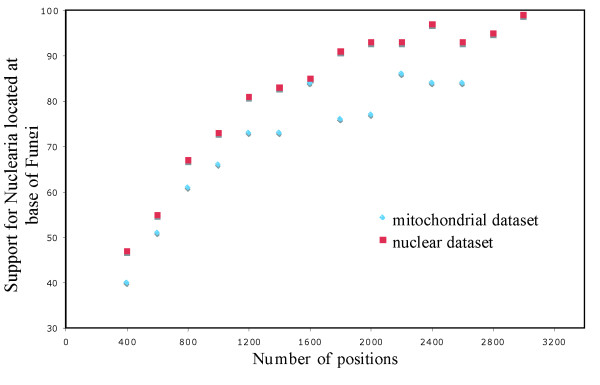
**VLB analysis**. Relationship between the number of sequence positions and bootstrap support for Fungi+Nucleariida, with nuclear and mitochondrial datasets.

Phylogenetic analysis of a dataset with 56 species and 13 of the ubiquitous, most conserved mtDNA-encoded proteins predicts the monophyly of Opisthokonta, Stramenopila, Holozoa and Fungi with confidence, and also recovers *Nuclearia *as the sister-group of Fungi, albeit with a moderate BS value of 85% (Figure 2). To verify if the limited support for Holomycota is expected (i.e., correlating with the number of available sequence positions in the respective datasets), we performed a variable length bootstrap (VLB) analysis. It compares the development of BS values with the number of sequence positions, for the nucleariid/fungal sister relationship. For this, we chose the 29 species shared between the two datasets (for the tree topology of the respective nuclear dataset see additional file 5). The results show that the development of BS values is similar for nuclear and mitochondrial data (Figure 3), and that the available mitochondrial dataset (as well as the above-cited nuclear phylogenies with five genes) is too small to resolve the phylogenetic position of nucleariids with high confidence. A better taxon sampling primarily in nucleariids will be imperative for improved phylogenetic resolution, motivating sequencing projects with new technologies, which are likely to provide mitochondrial as well as nuclear genome sequences - even with the limited amount of cellular material that is available for some taxa (e.g., [47]).

### Fungal phylogeny with Nucleariida as outgroup

**Figure 4:**
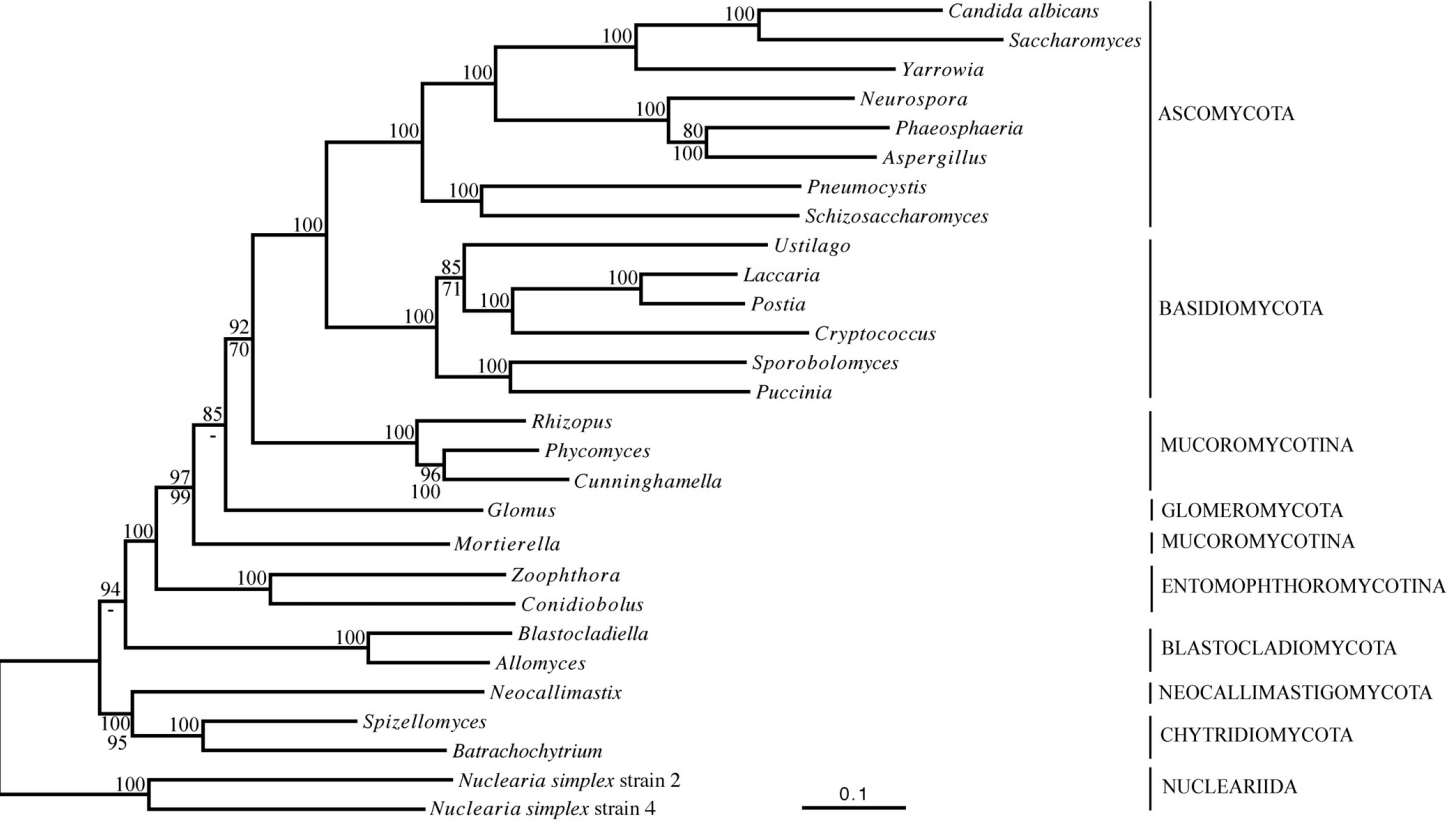
**Fungal phylogeny with nuclear data, using Nucleariida as the outgroup**. For details on figure description, evolutionary models and phylogenetic methods, see legend of Figure 1. Note that the phylogenetic position of Blastocladiomycota is unstable, differing between ML *versus *BI analyses (we consider the latter to be more reliable).

Analyses of both the nuclear and mitochondrial datasets have been insufficient to assess with confidence, neither zygomycete mono/paraphyly, nor the phylogenetic position of Blastocladiomycota (Blastocladiales) (Figure 1, 2). For instance, a recent mitochondrial multi-gene phylogeny with the first complete *Glomus *mtDNA sequence groups *Glomus *and *Mortierella*, yet lacks significant statistical support [47]. To re-address these questions, we have assembled a large dataset of nuclear-encoded genes from an extended, representative selection of fungal species, plus the two *Nuclearia *species as outgroup (i.e., the Fungal Dataset). The analyses show overall strong BS for the paraphyly of zygomycetes (Figure 4), i.e., the Entomophthoromycotina represent a significantly supported and completely independent fungal lineage. However, monophyletic Mucoromycotina including *Mortierella *as recently redefined [28] is not recovered (rendering the taxon Mucoromycotina paraphyletic), neither is the taxon Symbiomycota (Glomeromycota plus Dikarya; [29]). Instead, there is moderate support to group Mucorales plus Dikarya (92% BS in BI) and *Glomus *as their next neighbor (85% BS in BI). Although the placement of *Glomus *relative to *Mortierella *differs between our BI and ML analyses (Figure 4), we assume that the result of the BI analysis with its superior evolutionary model is more reliable. In light of these results, taxonomic reordering based on stable phylogenetic resolution of the traditional zygomycetes will require phylogenomic analyses with a much improved taxon sampling. Currently, nuclear and mitochondrial genome data are available only for single species in the latter two taxa; i.e. *Glomus intraradices *and *M. verticillata*.

Rooting of the fungal tree with nucleariids confirms that the traditional chytridiomycetes are also paraphyletic, again assuming that the result of the BI analysis is correct (Figure 4). Confirmation of this result (justifying an elevation of Blastocladiomycota as a separate phylum; [28]) is highly desirable, as genome-size datasets in Blastocladiomycota are limited to the two moderately distant species *Blastocladiella emersonii *and *A. macrogynus*. Similarly, in light of the significant support for a monophyletic Chytridiomycota plus Neocallimastigomycota (100% BS with BI; Figure 4), their division into separate taxonomic higher ranks should be reconsidered, but only after phylogenomic analysis with improved taxon sampling in both groups. Finally, our results motivate genome or EST sequencing in *Rozella *species (Rozellida), potential relatives of Microsporidia and close neighbors of Fungi. The availability of a largely improved taxon sampling in zygomycetes, chytrids and Rozellida will provide a solid basis for evaluating the proposed placements of Microsporidia - either within or as a sistergroup to Fungi - based on phylogenomic analyses.

The results presented here are consistent with previous notions on how Fungi came into being. For example it is thought that the first Fungi probably had branched chytrid-like rhizoids, which developed by enclosure of nucleariid-like filopodia (sometimes branched) into cell walls, during a nutritional shift from phagotrophy to saprotrophy, thus giving rise to fungal hyphae and rhizoids [7]. However, the picture is more complicated as it is widely thought that the ancestral opisthokont also had a single posterior flagellum [48]. This structure was lost during evolution of most but not all fungal lineages (e.g., [9,25,49,50]), with a separate loss in the nucleariid sistergroup. In this sense, nucleariids are unlikely to represent a primitive developmental stage, but rather a secondary reduction resulting in a unicellular, amoeboid life style. Obviously, the clarification of the chain of events leading to the emergence of multicellularity in Fungi is by no means complete. These issues will only become clear with a much broader sampling of genomes from taxa near the animal-fungal divergence and the discovery of additional protist groups that are closely related to Fungi.

## Conclusion

Here we demonstrate that phylogenomic analysis with improved evolutionary models and algorithms has a potential for resolving long-standing issues in fungal evolution, by increasing phylogenetic resolution. Yet, while our results support certain aspects of the new taxonomic classification of Fungi they contradict others, suggesting that the introduction of certain higher-level taxa is only preliminary. In particular, the elevation of Neocallimastigales, Blastocladiomycota and Glomeromycota to separate phyla is questionable from a molecular phylogenetics standpoint, and potentially confusing to the larger scientific community. At present, genome analyses continue to suffer from poor sampling in chytrids, zygomycetes and close fungal relatives such as nucleariids. This issue will be resolved by the employment of new, increasingly inexpensive genome sequencing technologies. Phylogenomic projects like the current one will help focusing on genome analyses of poorly known phyla and taxa that are key to understanding fungal origins and evolution.

## Methods

### Construction of cDNA libraries and EST sequencing

Two *N. simplex *(CCAP 1552/2 and 1552/4) cDNA libraries were constructed following recently published protocols [51]. Cells were grown in liquid standing cultures in WCL medium http://megasun.bch.umontreal.ca/People/lang/FMGP/methods/wcl.html supplemented with 0.5 × Cerophyll, with *E*. *coli *cells as food, which were pre-grown on LB medium in Petri-dishes as food. Plasmids were purified using the QIAprep 96 Turbo Miniprep Kit (Qiagen), sequencing reactions were performed with the ABI Prism BigDye™ terminator version 3.0/3.1 (Perkin-Elmer, Wellesley, MA, USA) and sequenced on an MJ BaseStation (MJ Research, USA). Trace files were imported into the TBestDB database http://tbestdb.bcm.umontreal.ca/searches/login.php[52] for automated processing, including assembly as well as automated gene annotation by AutoFact [53,54].

### Mitochondrial sequencing and genome annotation

*N. simplex *(CCAP 1552/2) was grown as described above. The harvested cells were disrupted by addition of SDS plus proteinase K, and mitochondrial DNA was purified following a whole cell lysate protocol [55] and sequenced from a random clone library [56]. For mitochondrial genome assembly we used Phred, Phrap and Consed [57,58]; http://www.phrap.org/. Mitochondrial genes and introns were identified using automated procedures (MFannot, N. Beck and BFL unpublished; RNAweasel, [59]), followed by manual curation of the results.

### Dataset construction

A previously published alignment of nuclear-encoded proteins [20] was used for adding the new *Nuclearia *cDNA sequences generated in our lab, plus extra sequences available from GenBank (a taxonomic broad dataset containing 50 eukaryotes will be referred to as the 'Eukaryotic Dataset'; another one containing 26 fungal species plus the two *Nuclearia *species as 'Fungal Dataset') using MUST [60] and FORTY (Denis Baurain and HP, unpublished). The number of species has been limited (to allow phylogenomic analyses within reasonable time frames), but only in well-sampled phylogenetic groups of undisputed phylogenetic affinity. Species that were not included are either fast-evolving and/or are incompletely sequenced. Other procedures for dataset construction, in particular the elimination of paralogous proteins, have been described previously [61]. Within opisthokonts, major lineages had to be represented by at least two distant species, and the extremely fast-evolving Microsporidia were excluded, as these are known to introduce phylogenetic artifacts and an overall reduction of phylogenetic resolution (at an extreme leading to misplacement of species; e.g., [62,63]). Sampling within the protist outgroup of the Eukaryotic Dataset is also not comprehensive (Stramenopila, Alveolata, and Euglenozoa) and limited to slow-evolving representatives of major eukaryotic lineages. The final Eukaryotic Dataset contains 118 proteins (24 439 amino acid positions) and the Fungal Dataset 150 proteins (40 925 amino acid positions). Proteins included in the nuclear datasets are listed in additional file 6.

For a dataset of mitochondrial proteins, 13 ubiquitous genes (*cox1, 2, 3, cob, atp6, 9*, and *nad1, 2, 3, 4, 4L, 5, 6*) were selected. Muscle ([64]), Gblocks ([65]) and an application developed in-house (mams) were used for automatic protein alignment, removal of ambiguous regions and concatenation. The final dataset contains 56 taxa and 2 710 amino acid positions.

### Phylogenetic analysis

Phylogenetic analyses were performed at the amino acid (aa) level using methods that are known to be least sensitive to LBA artifacts ([36,37,66], and references therein). The concatenated protein datasets were analyzed either by Bayesian inference (BI, PhyloBayes [37]) with the CAT+ Γ model and four discrete gamma categories, or by maximum likelihood (ML, RAxML [40] with the WAG+ Γ model and four discrete categories. BI analyses using the CAT model have been shown to be more reliable than ML, due to the application of a more realistic evolutionary model. ML analyses were essentially performed to identify differences in topology, pinpointing problematic parts of the tree for which addition of new data would be in order (i.e., preferentially genome sequences from slowly-evolving species, and those that are expected to break long internal branches at questionable tree topologies).

In case of BI and the Eukaryotic Dataset (values for the Fungal Dataset in brackets), chains were run for 3000 (1000) cycles, and the first 1500 (500) cycles were removed as burn-in corresponding to approximately 1,200,000 (400,000) generations. Convergence was controlled by running three independent chains, resulting in identical topologies. The reliability of internal branches for both, ML and BI analyses was evaluated based on 100 bootstrap replicates. For BI, we inferred a consensus tree from the posterior tree topologies of replicates.

Likelihood tests of competing tree topologies were also performed. The site-wise likelihood values were estimated using Tree-Puzzle [67] with the WAG+ Γ model, and p-values for each topology were calculated with CONSEL [45].

### Variable Length Bootstrap analysis

We compared the performance of nuclear and mitochondrial datasets in phylogenetic inference by Variable Length Bootstrap (VLB) analysis [68]. Sequences of 29 common species were taken from the eukaryotic (24,439 aa positions) and mitochondrial (2,710 aa positions) datasets. From these, two respective series of sub-datasets were constructed by randomly choosing 400, 600, 800, 1 000 ... sequence positions. Phylogenetic inferences were then performed using RAxML with the WAG+Γ model and four discrete categories, after which the BS values for the grouping of nucleariids and Fungi were recorded.

## Authors' contributions

ETS and LF constructed and sequenced the two Nuclearia EST libraries; YL, HB, HP and BFL conducted phylogenetic analyses. All authors participated in writing, reading and approving the manuscript.

## Supplementary Material

Additional file 1 **Tree of eukaryotes with the Eukaryotic Dataset and ML inference**. The analyses using RAxML (WAG+Gamma; four categories) support the grouping of Malawimonadozoa and JEH with a BS of 77%. Other differences with the tree using BI method (Figure 1) include Plantae relationships, and the placement of Haptophyceae, which receive no support for both BI and ML analyses with this dataset. For more details see legend of Figure 1. Click here for file

Additional file 2 **Phylogeny with Eukaryotic Dataset after removing fast-evolving species**. The tree was inferred with ML (RAxML) using the WAG+Gamma model with four categories. Numbers at branches represent support values obtained with 100 bootstrap replicates. Click here for file

Additional file 3 **Phylogeny with Eukaryotic Dataset after recoding amino acids into six groups**. The amino acids of the Eukaryotic Dataset were recoded into six groups as follows: (1) ASTGP, (2) DNEQ, (3) RKH, (4) MVIL, (5) FYW and, (6) C. This allowed the use of a 6 × 6 general time-reversible rate matrix with free parameters rather than a fixed empirical matrix. Sequence composition and among-site rate variation parameters were also free in the BI analysis, as implemented in P4 (Foster PG: Modeling compositional heterogeneity. *Syst Biol *2004, **53**(3):485-495); 50 000 generations, first 10 000 removed as burn-in). Numbers at branches represent PP values. Click here for file

Additional file 4 **Complete mitochondrial DNA (mtDNA) of *Nuclearia simplex *strain 1552/2**. The circular-mapping mtDNA (74,120 bp) is displayed starting with the *rnl *gene (coding for the large subunit rRNA), clockwise in direction of transcription. Black bars, genes or exons; grey bars, introns and intronic ORFs; tRNA genes are named by the one-letter amino acid code. Click here for file

Additional file 5 **Phylogenetic relationship of the 29 species used for VLB analyses**. The phylogeny was inferred using nuclear data and RAxML with the WAG + Gamma model. In order to minimize missing data in the mitochondrial dataset, we exchanged *Saccharomyces *and *Schizosaccharomyces *that both lost the six mitochondrion-encoded *nad *genes with *Candida albicans *and *Taphrina*. Click here for file

Additional file 6 **Proteins included in phylogenetic datasets**. List of proteins included in the Eukaryotic and Fungal Datasets. Click here for file
