# Supplementary figures and images for: Phylogenomic analyses predict sistergroup relationship of nucleariids and Fungi and paraphyly of zygomycetes with significant support
Source: BMC Evol Biol. 2009 Nov 25;9:272. doi: 10.1186/1471-2148-9-272 (PMC2789072; doi:10.1186/1471-2148-9-272)

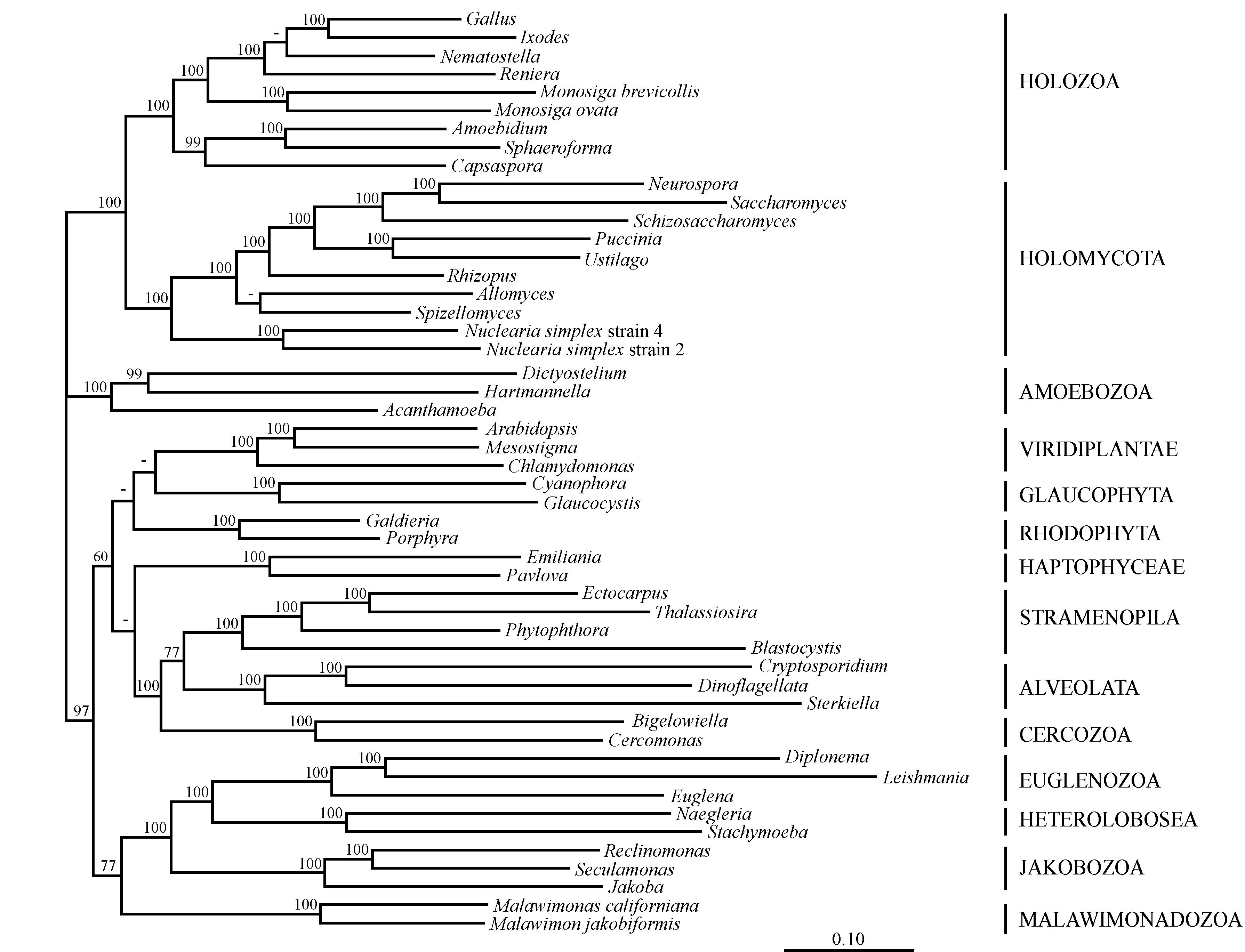

Supplement: Additional file 1 — Tree of eukaryotes with the Eukaryotic Dataset and ML inference. The analyses using RAxML (WAG+Gamma; four categories) support the grouping of Malawimonadozoa and JEH with a BS of 77%. Other differences with the tree using BI method (Figure 1) include Plantae relationships, and the placement of Haptophyceae, which receive no support for both BI and ML analyses with this dataset. For more details see legend of Figure 1. [file 1471-2148-9-272-S1.DOC]

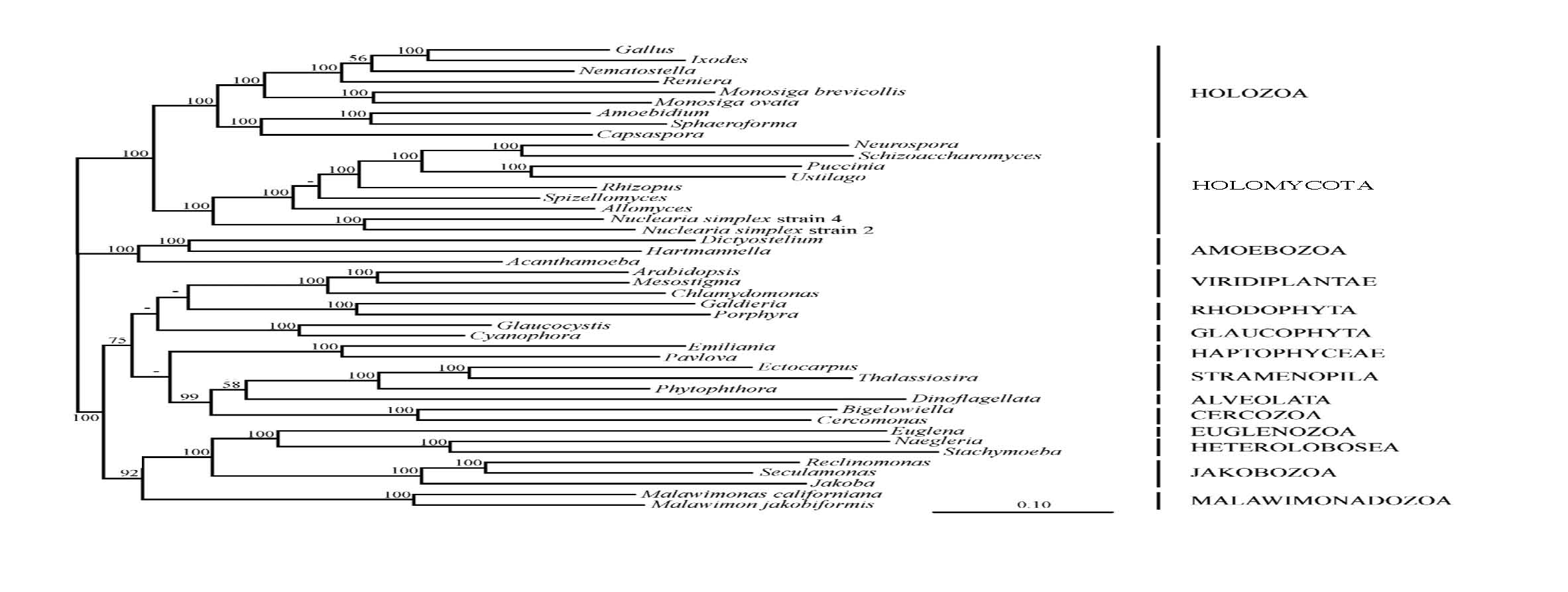

Supplement: Additional file 2 — Phylogeny with Eukaryotic Dataset after removing fast-evolving species. The tree was inferred with ML (RAxML) using the WAG+Gamma model with four categories. Numbers at branches represent support values obtained with 100 bootstrap replicates. [file 1471-2148-9-272-S2.DOC]

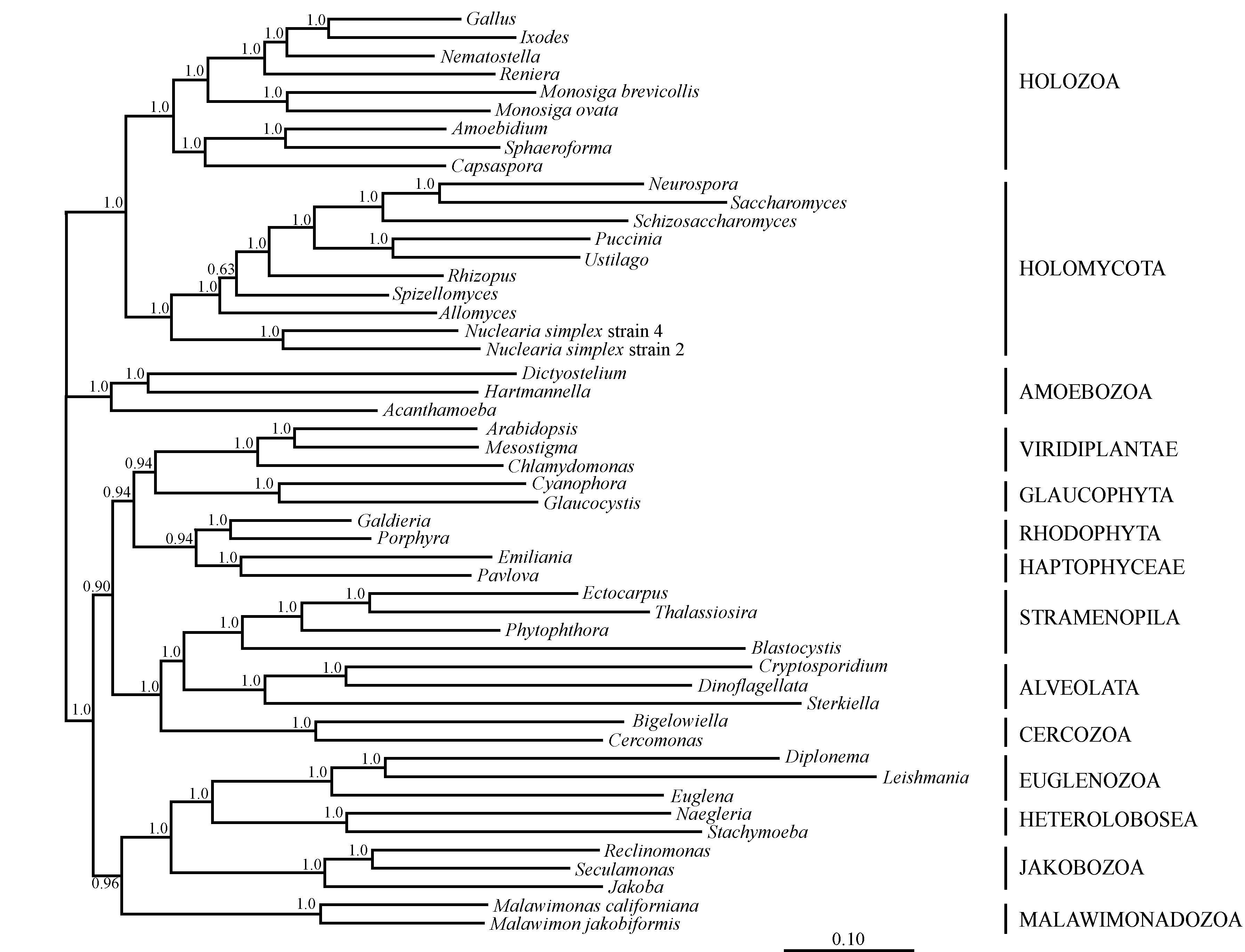

Supplement: Additional file 3 — Phylogeny with Eukaryotic Dataset after recoding amino acids into six groups. The amino acids of the Eukaryotic Dataset were recoded into six groups as follows: (1) ASTGP, (2) DNEQ, (3) RKH, (4) MVIL, (5) FYW and, (6) C. This allowed the use of a 6 × 6 general time-reversible rate matrix with free parameters rather than a fixed empirical matrix. Sequence composition and among-site rate variation parameters were also free in the BI analysis, as implemented in P4 (Foster PG: Modeling compositional heterogeneity. Syst Biol 2004, 53(3):485-495); 50 000 generations, first 10 000 removed as burn-in). Numbers at branches represent PP values. [file 1471-2148-9-272-S3.DOC]

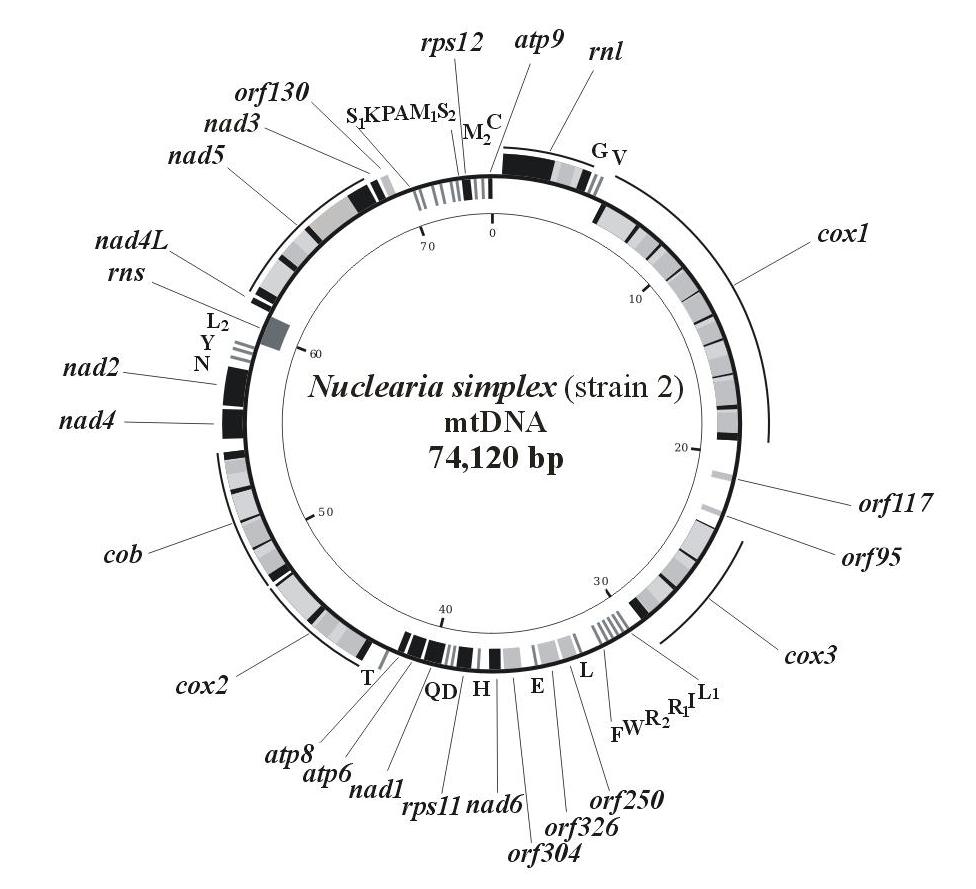

Supplement: Additional file 4 — Complete mitochondrial DNA (mtDNA) of Nuclearia simplex strain 1552/2. The circular-mapping mtDNA (74,120 bp) is displayed starting with the rnl gene (coding for the large subunit rRNA), clockwise in direction of transcription. Black bars, genes or exons; grey bars, introns and intronic ORFs; tRNA genes are named by the one-letter amino acid code. [file 1471-2148-9-272-S4.DOC]

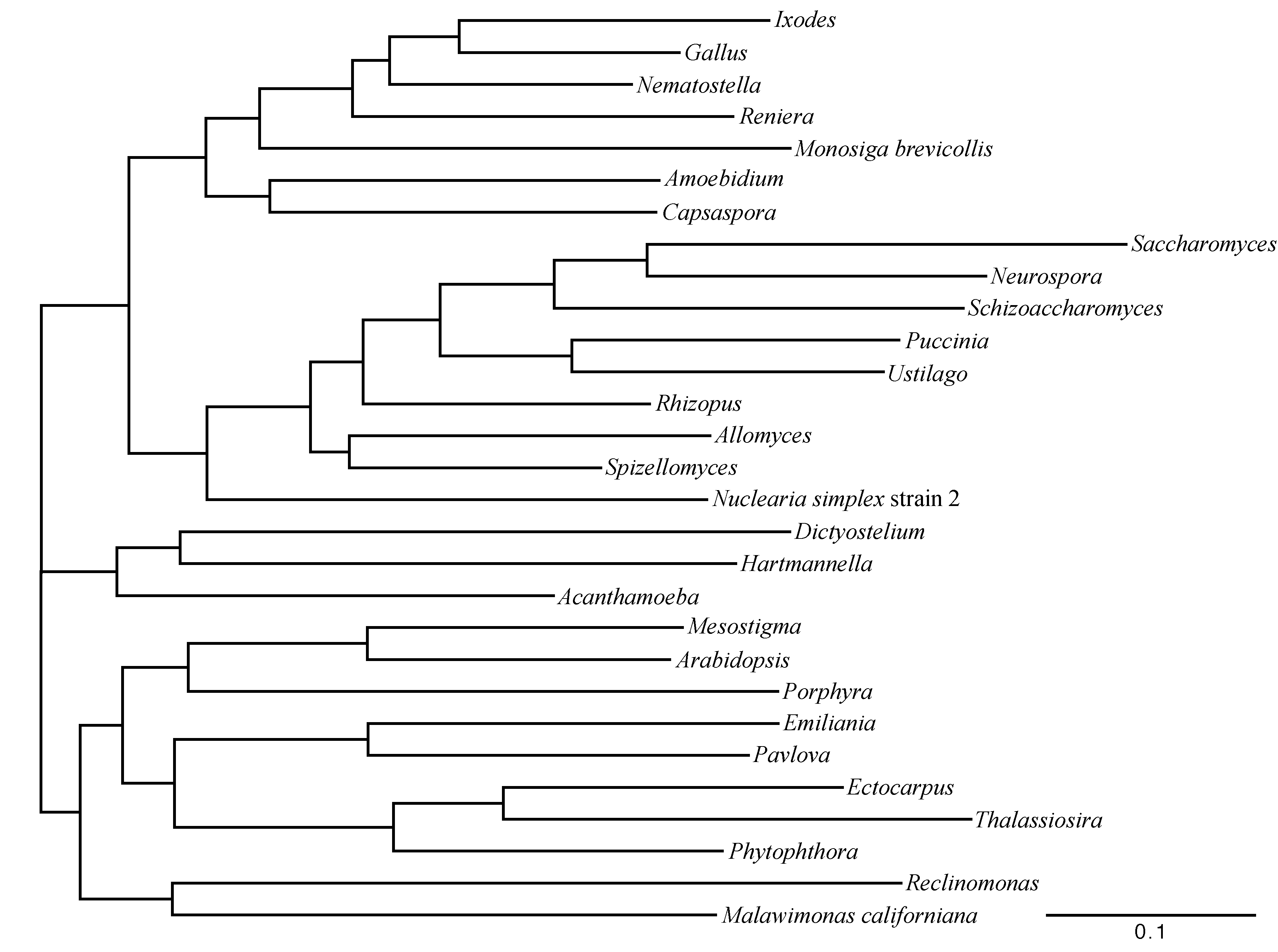

Supplement: Additional file 5 — Phylogenetic relationship of the 29 species used for VLB analyses. The phylogeny was inferred using nuclear data and RAxML with the WAG + Gamma model. In order to minimize missing data in the mitochondrial dataset, we exchanged Saccharomyces and Schizosaccharomyces that both lost the six mitochondrion-encoded nad genes with Candida albicans and Taphrina. [file 1471-2148-9-272-S5.DOC]
