# Supplementary material for: Phylogenomic analyses predict sistergroup relationship of nucleariids and Fungi and paraphyly of zygomycetes with significant support
Source: BMC Evol Biol. 2009 Nov 25;9:272. doi: 10.1186/1471-2148-9-272 (PMC2789072; doi:10.1186/1471-2148-9-272)
Supplement: Additional file 6 — Proteins included in phylogenetic datasets. List of proteins included in the Eukaryotic and Fungal Datasets. [file 1471-2148-9-272-S6.DOC]

**Table S1**: Proteins in the Eukaryotic Dataset

| arp23 | cct-A | cct-B | cct-D | cct-E | cct-G | cct-N | cct-T |
| --- | --- | --- | --- | --- | --- | --- | --- |
| cct-Z | cpn60-mt | ef1-EF1 | ef1-RF3 | ef2-EF2 | fibri | grc5 | if1a |
| if2b | if2g | if6 | ino1 | l12e-A | l12e-C | l12e-D | nsf1-C |
| nsf1-G | nsf1-I | nsf1-J | nsf1-K | nsf1-L | nsf1-M | nsf2-A | psma-A |
| psma-B | psma-C | psma-D | psma-E | psma-F | psma-G | psmb-H | psmb-I |
| psmb-J | psmb-K | psmb-L | psmb-M | psmb-N | rad51-A | rf1 | rpl1 |
| rpl11b | rpl12b | rpl13 | rpl14a | rpl15a | rpl16b | rpl17 | rpl18 |
| rpl19a | rpl2 | rpl20 | rpl21 | rpl22 | rpl23a | rpl24-A | rpl25 |
| rpl26 | rpl27 | rpl3 | rpl30 | rpl31 | rpl32 | rpl33a | rpl34 |
| rpl35 | rpl37a | rpl38 | rpl39 | rpl42 | rpl43b | rpl4B | rpl5 |
| rpl6 | rpl7-A | rpl9 | rpp0 | rps1 | rps10 | rps11 | rps13a |
| rps14 | rps15 | rps16 | rps17 | rps18 | rps19 | rps2 | rps20 |
| rps22a | rps23 | rps25 | rps26 | rps27 | rps28a | rps29 | rps3 |
| rps4 | rps5 | rps6 | rps8 | sap40 | srp54 | srs | suca |
| vata | vatb | vatc | vate | w09c |  |  |  |

**Table S2**: Proteins in the Fungal Dataset

| arc20 | arp23 | cct-A | cct-B | cct-D | cct-E | cct-G | cct-N |
| --- | --- | --- | --- | --- | --- | --- | --- |
| cct-T | cct-Z | cpn60-mt | crfg | ef1-EF1 | ef1-RF3 | ef2-EF2 | ef2-U5 |
| eif5a | er1 | fibri | fpps | grc5 | hsp70-E | hsp70-mt | hsp90-C |
| if1a | if2b | if2g | if2p | if4a-a | if4a-b | if6 | ino1 |
| l12e-A | l12e-B | l12e-C | l12e-D | mcm-B | mcm-C | mcm-E | mcm-F |
| nsf1-G | nsf1-J | nsf1-K | nsf1-L | nsf1-M | nsf2-A | pace2-A | pace2-C |
| psma-A | psma-B | psma-C | psma-D | psma-E | psma-F | psma-G | psmb-H |
| psmb-J | psmb-K | psmb-L | psmb-M | psmb-N | rad23 | rad51-A | rf1 |
| rpl1 | rpl11b | rpl12b | rpl13 | rpl14a | rpl15a | rpl16b | rpl17 |
| rpl18 | rpl19a | rpl2 | rpl20 | rpl21 | rpl22 | rpl23a | rpl24-A |
| rpl25 | rpl26 | rpl27 | rpl3 | rpl30 | rpl31 | rpl32 | rpl33a |
| rpl34 | rpl35 | rpl36 | rpl37a | rpl38 | rpl39 | rpl42 | rpl43b |
| rpl4B | rpl5 | rpl6 | rpl7-A | rpl9 | rpo-A | rpo-B | rpo-C |
| rpp0 | rps1 | rps10 | rps11 | rps13a | rps14 | rps15 | rps16 |
| rps17 | rps18 | rps19 | rps2 | rps20 | rps22a | rps23 | rps24 |
| rps25 | rps26 | rps27 | rps27a | rps28a | rps29 | rps3 | rps4 |
| rps5 | rps6 | rps7 | rps8 | rps9 | sap40 | srp54 | srs |
| suca | tfiid | tif2a | vata | vatb | vate | xpb | vatpased |
| ATP synthase-mt | | dihydrolatransacylase-b | | | ornamtrans-a | |  |
| pyrdehydroe1b-mt | | sadhchydrolase-E1 | | | vacaatpasepl21-a | |  |
